# Differential diagnosis and laparoscopic resection of an adrenal pseudocyst: A case report

**DOI:** 10.1016/j.ijscr.2020.05.082

**Published:** 2020-06-09

**Authors:** Yuichiro Yokoyama, Yusuke Tajima, Izuru Matsuda, Kentaro Kamada, Takashi Ikehara, Toshimasa Uekusa, Hirokazu Momose, Satomi Yoneyama, Hiroki Sakata, Akio Hidemura, Hiroyuki Suzuki, Masahiro Ishimaru

**Affiliations:** a Department of Surgery, Douai Memorial Hospital, 2-1-11 Yokoami, Sumida-ku, Tokyo, 130-8587, Japan; b Department of Surgery, Kanto Rosai Hospital, 1-1 Kizukisumiyoshicho, Nakahara-ku, Kanagawa, 211-8510, Japan; c Department of Radiology, Kanto Rosai Hospital, 1-1 Kizukisumiyoshicho, Nakahara-ku, Kanagawa, 211-8510, Japan; d Department of Gastrointestinal Medicine, Kanto Rosai Hospital, 1-1 Kizukisumiyoshicho, Nakahara-ku, Kanagawa, 211-8510, Japan; e Department of Pathology, Kanto Rosai Hospital, 1-1 Kizukisumiyoshicho, Nakahara-ku, Kanagawa, 211-8510, Japan

**Keywords:** CT, Computed tomography, MRI, Magnetic resonance imaging, EUS, endoscopic ultrasonography, Adrenal cyst, Pancreatic cyst, Laparoscopic resection

## Abstract

**Background:**

Adrenal pseudocysts are infrequent entities and definite preoperative diagnosis is difficult. We present a case of left adrenal pseudocyst, which was intraoperatively identified as having an adrenal origin and was resected using a laparoscopic approach.

**Presentation of case:**

A 41-year-old female was referred to our hospital for examination and treatment of a cystic lesion in the pancreatic tail. Preoperative diagnostic imaging studies showed a cystic lesion with intramural nodular structure, measuring 39 mm in the largest diameter and located between the pancreatic tail and the left adrenal gland. However, the origin of the cystic lesion remained unclear, and a definite preoperative diagnosis was not established. The cystic lesion was intraoperatively identified as having an adrenal origin after the division of the loose connective tissue layer around the lesion under the laparoscopic magnified view. Laparoscopic left adrenalectomy was performed as radical treatment and the histopathological diagnosis confirmed the presence of an adrenal pseudocyst.

**Discussion:**

We could not ascertain the origin of the cystic lesion from the left adrenal gland and establish a definite diagnosis based on the findings of the preoperative diagnostic imaging modalities. Laparoscopic surgery could be more advantageous than the conventional open approach as not only a minimally invasive treatment option but also as an intraoperative diagnostic tool for cystic lesions in the pancreatic tail.

**Conclusion:**

This case report suggests that laparoscopic surgery could be clinically useful as not only a minimally invasive treatment but also an intraoperative diagnostic tool for cystic lesions in the pancreatic tail region.

## Highlights

• Adrenal pseudocysts are infrequent entities.
• The preoperative diagnosis of left adrenal pseudocysts is sometimes difficult.
• Laparoscopic surgery is useful as an adrenal cyst intraoperative diagnostic tool.

## 1 Introduction

Adrenal pseudocysts are infrequent entities, and the majority of them are benign cystic masses that are enclosed by a fibrous wall [1]. Their pathogenesis is considered to be based on repeated episodes of trauma, infection, or bleeding [2]. Asymptomatic, nonfunctioning, benign-appearing adrenal cysts may be followed up without surgical treatment. A major issue about their management is their preoperative diagnosis, which renders subsequent management difficult, as imaging modalities often fail to determine their exact origin. In particular, left adrenal pseudocysts have been shown to mimic cystic lesions of the pancreas, liver, kidney, retroperitoneum, and spleen [1,[3], [4], [5]]. We present a case of a patient with a left adrenal pseudocyst, which was intraoperatively identified as having an adrenal origin and was laparoscopically resected. To the best of our knowledge, reports describing the clinical usefulness of laparoscopic surgery as not only a minimally invasive treatment but also as an intraoperative diagnostic tool for retroperitoneal cystic lesions are rare. The work has been reported in line with the SCARE criteria [6].

## 2 Presentation of case

**Fig. 1:**
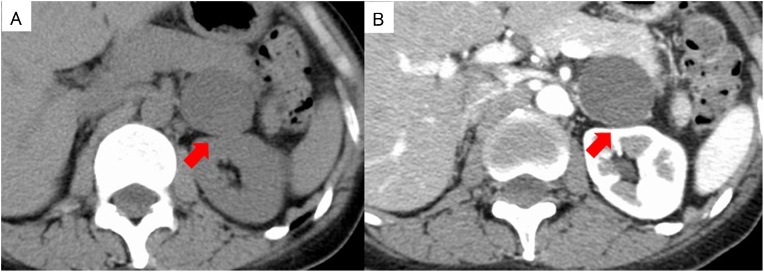
Computed tomography (CT) revealing a homogenous unilocular cystic mass between the pancreatic tail and the left adrenal grand, measuring 39 mm in the largest diameter (red arrow) (A). The wall of the cystic lesion is slightly enhanced and there are no solid components or calcification (red arrow) (B).

**Fig. 2:**
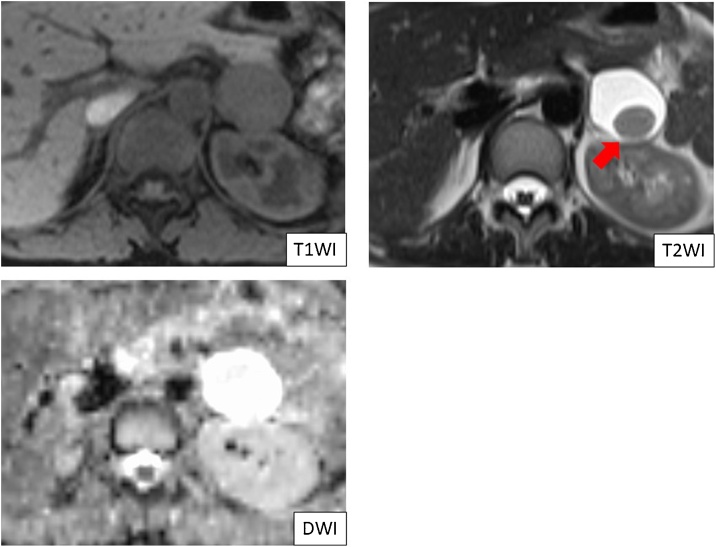
Magnetic resonance imaging. The cystic lesion shows low intensity in T1-weighted images, high intensity in T2-weighted images, and high intensity in diffusion-weighted images. In T2-weighted images, a round-shaped low intensity area, measuring 11 mm, was detected in the cystic lesion.

**Fig. 3:**
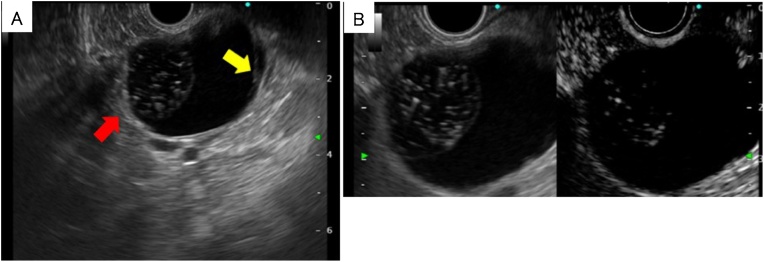
Endoscopic ultrasonography (EUS) revealing a cystic lesion in the region of the pancreatic tail and suggestive of internal septa (red arrow) and intramural nodular structure showing mixed low- and high-echoic areas (yellow arrow) (A). Enhanced EUS detected no enhancement in the whole lesion (B).

A 41-year old female had undergone a medical examination, which revealed a cystic lesion in the pancreatic tail region on abdominal ultrasonography. She was referred to our hospital for further examination and treatment. She had no relevant medical history and no family history of pancreatic disease. On physical examination, her abdomen was flat and no abdominal mass was detected. Vital signs were stable and routine laboratory tests were within normal limits, including tumor markers. The hormonal examination, which included serum catecholamines, cortisol, aldosterone, adrenocorticotropic hormone, dehydroepiandrosterone, plasma renin activity, and urinary catecholamine and metanephrine levels were all within normal limits. Computed tomography (CT) showed a homogenous unilocular cystic mass in the pancreatic tail region, measuring 39 mm in the largest diameter (Fig. 1A). The cystic lesion was located between the pancreatic tail and the left adrenal gland. However, the border between the cystic lesion and both the pancreas and adrenal gland was obscure. The wall of the cystic lesion was slightly enhanced, and there were no solid components and calcification (Fig. 1B). Magnetic resonance imaging (MRI) revealed that the cystic lesion contents showed high intensity in T2-weighted images, low intensity in T1-weighted images, and high intensity in diffusion-weighted images (Fig. 2). In T2-weighted images, a round-shaped low intensity area, measuring 11 mm, was also detected (Fig. 2). There was no connection between the pancreatic duct and the cyst on magnetic resonance cholangiopancreatography. Endoscopic ultrasonography (EUS) revealed a cystic lesion in the pancreatic tail region and suggested the presence of internal septa and an intramural nodular structure showing low- and high- mixed echoic areas (Fig. 3A). EUS did not detect the left adrenal gland. Enhanced EUS also found no enhancement of the whole lesion (Fig. 3B). Based on these observations, the preoperative suspected diagnosis was either lymphoepithelial cyst, lymphangioma, pseudocyst, or foregut cyst of the pancreas, adrenal gland, or retroperitoneum. In addition, the possibility of cystic neoplasms with malignant potential, such as mucinous cystic neoplasm of the pancreas (or retroperitoneum), could not be ruled out. The origin of the cystic lesion remained ambiguous and the definite preoperative diagnosis was considered unclear. Therefore, laparoscopic transabdominal resection was undertaken to provide a definite diagnosis and treatment.

**Fig. 4:**
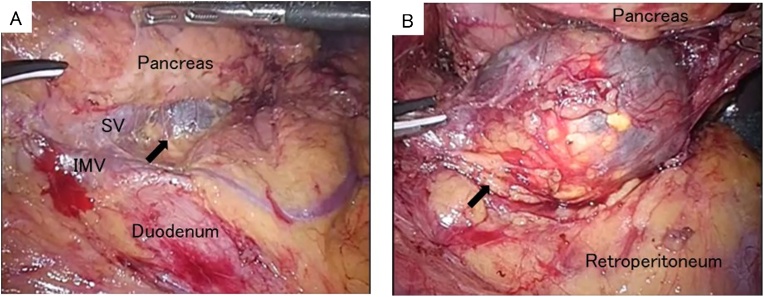
Laparoscopic view. Black arrow indicates a cystic lesion under the pancreatic tail (A). Black arrow indicates the cystic lesion of the left adrenal gland (B). SV, splenic vein; IMV, inferior mesenteric vein.

**Fig. 5:**
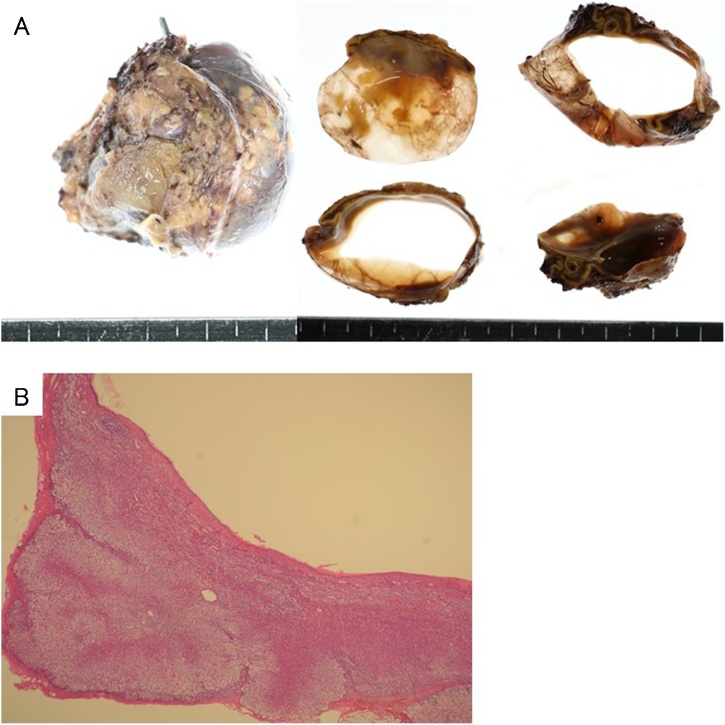
Gross appearance of the resected specimen revealing an unilocular cystic lesion, measuring 42 × 40 × 32 mm (A). Histopathologically, the cystic wall consisted of fibrocollagenous tissue without endothelial and epithelial lining (H&E, original magnification ×40) (B).

Under general anesthesia, 5 trocars, including 3 12-mm trocars and 2 5-mm trocars, were inserted. The omentum was incised and the omental bursa was opened. The splenic flexure of the colon was mobilized from the left paracolic gutter and the inferior pole of the spleen. The splenocolic and splenorenal ligaments were divided to expose the left retroperitoneum. A cystic mass was detected adjacent to and under the pancreatic tail (Fig. 4A). Pulling the pancreas tail upward, the loose connective tissue layer around the cystic lesion was carefully and precisely dissected using HARMONIC ® HD 1000i shears (Ethicon Endo-Surgery, Cincinnati, OH). After the dissection around the cystic lesion, we identified that the cystic lesion originated from the left adrenal gland (Fig. 4B); laparoscopic left adrenalectomy was then performed as radical treatment. The operative time was about 2 h and 20 min with minimal blood loss. Postoperatively, the patient made an uncomplicated recovery and was discharged on day 6. The gross appearance of the surgically resected specimen revealed that the cystic lesion was unilocular, measuring 42 × 40 × 30 mm (Fig. 5A). The content of the cystic lesion was light brown serous fluids, while internal septa and intramural nodular structure were not recognized. On the histopathological examination, the cystic wall consisted of fibrocollagenous tissue without endothelial and epithelial lining (Fig. 5B). There was no evidence of malignancy. The histopathological diagnosis was confirmed to be adrenal pseudocyst.

## 3 Discussion

Adrenal cysts are rare entities, mainly diagnosed in autopsy studies in 0.06–0.18% of the population [7,8]. Adrenal cysts are roughly divided into 2 categories: true cysts and pseudocysts. True cysts are classified into 2 categories: endothelial (lymphatic or vascular) and epithelial, while pseudocysts are classified into 3 categories: hemorrhagic, neoplastic, and parasitic [2]. The majority of adrenal cysts are non-functional and asymptomatic and are found incidentally on imaging studies. By definition, adrenal pseudocysts consist of a fibrous wall without a cellular lining. The management of adrenal pseudocysts depends on some factors, such as the presence of symptoms, functional status, and the probability of malignancy [5]. The incidence of malignancy in adrenal cystic lesions is less than 7% and is mainly related to their size [9]. Surgical treatment is recommended by the presence of symptoms and for cysts larger than 5 cm or complicated ones with suspicion of malignancy [5]. Typically, radiological findings of adrenal pseudocysts reveal thin walls filled with watery fluid and occasional calcifications [10]. However, despite recent advances in diagnostic imaging modalities, such as EUS, CT, and MRI, adrenal pseudocysts remain difficult to distinguish preoperatively from cystic lesions of other organs, including the pancreas, due to the indistinct lesion margins and adhesions to adjacent organs [1,3,4]. In addition, adrenal pseudocysts are occasionally more complex and may have thick walls and internal septa, which makes them difficult to differentiate from pancreatic cystic lesions [9]. The frequency of pancreatic cystic lesions is known to be much higher than that of adrenal cysts [3,4,11,12]. In several previous reports, adrenal pseudocysts have been misinterpreted as a pancreatic lesion because of their similarity to a pancreatic tail cyst [3,4]. Recently, Voudoukis et al. reported that EUS is superior to all other imaging studies for the preoperative diagnosis of adrenal cystic lesions, especially when a pancreatic origin is hard to be ruled out by other diagnostic tools [3]. However, in our case, we could not ascertain the origin of the cystic lesion from the left adrenal gland and establish a definite diagnosis based on the findings of the preoperative diagnostic imaging modalities, including EUS. Therefore, it is essential that adrenal pseudocyst should be considered as a differential diagnosis when a retroperitoneal cystic lesion is seen in the pancreatic tail region. However, surgical excision would be a rational approach to establish a definite diagnosis, especially for patients with cystic lesions in which the malignant potential cannot be excluded. Since the introduction of laparoscopic transperitoneal adrenalectomy by Gagner et al. in 1992, surgery for adrenal lesions has gradually evolved from the open technique toward the laparoscopic approach as a minimally invasive method. Laparoscopic surgery permits small incisions, minimizes bowel manipulation, and decreases preoperative morbidity, leading to shorter hospital stays and faster recovery [13]. With respect to the surgical treatment of adrenal pseudocysts, some studies state that open resection is the preferred technique in patients with large-sized cysts (>6 cm), while the laparoscopic approach may be a valuable treatment option for cysts smaller than 6 cm [13]. However, recent literature on laparoscopic adrenalectomy suggests using the laparoscopic approach for adrenal pseudocysts irrespective of their size [1,5,14]. Our case report has shown that laparoscopic adrenalectomy is a safe option for the treatment of adrenal pseudocysts while maintaining the benefits of minimal invasiveness. Therefore, it is suggested that the laparoscopic approach should be considered the standard surgical approach to adrenal pseudocysts. In addition, our case report has also suggested the clinical usefulness of laparoscopic surgery as an intraoperative diagnostic tool for adrenal gland cystic lesions, which are difficult to distinguish from cysts of other organs, therefore helping to establish a definite diagnosis. Marwah et al. have emphasized that the diagnostic difficulty associated with adrenal pseudocysts increases the complexity of surgical treatment [4]. In fact, there have been several reports in which distal pancreatectomy was performed for adrenal cysts mimicking pancreatic cystic neoplasms using a conventional open approach [3,4]. In our case, after the division of the loose connective tissue layer around the lesion under the laparoscopic magnified view, the cystic lesion was intraoperatively identified as having an adrenal origin; laparoscopic adrenalectomy was then performed as radical treatment. Kim et al. reported an adrenal pseudocyst mimicking a retroperitoneal mucinous cystic neoplasm treated by laparoscopic resection [1]. They concluded that the laparoscopic approach should be the initial choice for such cystic lesions of the retroperitoneal organs. Laparoscopic magnified view enables us to identify small vessels, nerves, and fascial laminations that are invisible to the naked eye in conventional open surgery. As a result, precise dissection around the lesion under the laparoscopic view would help surgeons identify the exact origin of the lesion and make decisions about the appropriate surgical treatment for each patient. Therefore, we strongly believe that laparoscopic surgery could be more advantageous as not only a minimally invasive treatment option but also as an intraoperative diagnostic tool for cystic lesions in the pancreatic tail region as compared with the conventional open approach.

## 4 Conclusion

We presented a case of a patient with a left adrenal pseudocyst who was intraoperatively identified as having an adrenal origin and was laparoscopically resected. This case report suggests that laparoscopic surgery could be clinically useful as not only a minimally invasive treatment option but also as an intraoperative diagnostic tool for cystic lesions in the pancreatic tail region.

## Declaration of Competing Interest

The authors declare that they have no competing interests.

## Funding

There is no sources of funding for our research.

## Ethics approval

Treatments for the patient were in accordance with the ethical standards of the responsible committees on human experimentation (institution and national).

## Consent

Written informed consent was obtained from the patient for publication of this case report and accompanying images. A copy of the written consent is available for review by the Editor-in-Chief of this journal on request.

## Authors’ contributions

YY conceived the study, collected data, and drafted the manuscript. YT conceived the study, corrected and revised the manuscript. IM helped in the radiological diagnosis. KK and TI conducted EUS and helped in diagnosis. TU helped in histopathological diagnosis. HM, SY, HS, AH, HS, and MI corrected and revised the manuscript. All authors read and approved the final manuscript.

## Registration of research studies

Our case report is not first-in-man or animal studies. So, in accordance with guidance of research registry, we do not register our case report in http://www.researchregistry.com.

## Guarantor

Yuichiro Yokoyama.

## Provenance and peer review

Not commissioned, externally peer-reviewed.
